# Identification of Factors Leading to Damage of Semi-Elliptical Leaf Springs

**DOI:** 10.3390/ma18235426

**Published:** 2025-12-02

**Authors:** Mariusz Stańco, Marcin Kaszuba, Iwona Herbik

**Affiliations:** 1 Department of Machine and Vehicle Design and Research, Faculty of Mechanical Engineering, Wroclaw University of Science and Technology, Lukasiewicza 7/9, 50-371 Wrocław, Poland; 2 Center for Materials Engineering and Metal Forming, Faculty of Mechanical Engineering, Wroclaw University of Science and Technology, Lukasiewicza 5, 50-371 Wrocław, Poland; marcin.kaszuba@pwr.edu.pl; 3 Faculty of Mechanical Engineering, Wroclaw University of Science and Technology, Lukasiewicza 5, 50-371 Wrocław, Poland

**Keywords:** failure and fatigue leaf spring, 51CrV4, microhardness of leaf spring steel, experimental test of suspension and leaf spring, microstructure of leaf spring

## Abstract

This article presents the results of experimental investigations conducted to explain the causes of premature failure of two leaves of a semi-elliptical leaf spring mounted in a four-axle heavy-duty truck. The primary intended use of the vehicle was the continuous transport of cargo on unpaved roads with large, non-uniform irregularities. The vehicle equipped with the springs in question was loaded with a constant cargo placed in a rigid container. The Gross Vehicle Mass (GVM) was 32,000 kg (8000 kg/axle). During operation, it mostly traveled on rough terrain and off-road, at an average speed not exceeding 30 km/h. The semi-elliptical leaf springs used in the vehicle were supplied by a domestic manufacturer and produced according to a standard procedure that has been used for years. The experimental research included strain measurements of the springs during normal vehicle operation. In parallel, metallographic examinations of the fractured surfaces of the leaves were performed. The stress intensity (or stress state) of the springs in the vicinity of the resulting crack was also checked using the Finite Element Method (FEM). Subsequently, the fatigue life of the springs was estimated based on fatigue data available in the literature and the results of the conducted research.

## 1. Introduction

The harsh road conditions on which special-purpose vehicles must generally operate [1,2] require their designers to ensure that they are durable enough so that none of the components suffer premature degradation or unpredictable damage. The automotive sector is continuously evolving, as manufacturers focus on improving design efficiency, improving passenger safety, and reducing emissions. Under the influence of the above, key vehicle components are being redesigned and new production methods are being introduced [3]. Some of the most stressed assemblies in any car are components of the vehicle’s suspension system. They are the ones directly involved in transmitting forces from uneven pavement, ensuring that the vehicle moves safely. In addition, they ensure that loads caused by inertial forces generated during normal driving or sudden maneuvers are safely transferred to the vehicle’s wheels. In the case of trucks, suspension systems are built in the form of dependent systems, and in special vehicles produced mainly for the military, they are also built as independent systems. Air suspensions, as well as combinations of the three, are also encountered. Dependent suspension systems are mainly based on springs, which connect the drive axle to the vehicle frame to ensure easy vehicle handling as well as the transfer of forces from the wheels to the vehicle frame and vice versa. With independent suspensions, wheel displacement on one side does not affect wheel displacement on the other side. These systems are built with control arms supported by springs to ensure safe suspension operation. Of the aforementioned vehicle suspension types, the least complicated system is the dependent system, and it is the one most often used in truck construction. In the suspension, the component that is most stressed and on which fatigue life mainly depends is the leaf spring. The fatigue process determines the durability of most products, for example railway rails [4] and forging dies [5].

The history of the use of leaf springs in the first vehicles dates back to the mid-17th century, when leaf suspension was first used in horse-drawn carriages. The 18th century and Richard Lovell Edgeworth’s continuous development and research brought about their large-scale use in all types of horse-drawn vehicles. The use of leaf springs in the truck meant that the vehicle could carry large loads while maintaining vertical stability when traveling on both paved and dirt roadways, overcoming large terrain irregularities. Until the end of the 1970s, semi-elliptical springs were used extensively, and as a result of the increased carrying capacity of cars, they were supplanted by lighter parabolic springs, for which special manufacturing technology was developed to achieve satisfactory durability results with reduced tare weight. However, this is not sufficient, as the continuous development of technology, new manufacturing capabilities, ongoing searches for improved materials, and the optimization of load-bearing structures lead to the pursuit of increasingly modern solutions that enhance both the fatigue life and the immediate (static) strength of leaf springs, for example, through the use of composite materials [6], which also results in a significant reduction in their own weight [7]. To date, however, you can see many vehicles where semi-elliptical springs are still successfully used. The advantage of semi-elliptical springs is that when one of the leaves is damaged, the remaining leaves can carry the loads generated by the operation of the vehicle, and it is not necessary to replace the spring with a new one at the time the defect is noticed. Instead, the vehicle can be safely transported to a place where such a replacement can take place. Heat treatment has a significant impact on the fatigue properties and strength of steel [8,9,10].

Suspension components in heavy-duty vehicles operating under severe road conditions—such as on construction sites, in rough terrain during the construction of flood embankments, or other structures located away from public roads—are prone to fatigue cracking. This is mainly caused by the high stress amplitudes generated during operation, although cracks due to assembly errors or manufacturing defects can also occur [11,12,13].

## 2. Materials and Methods

### 2.1. Analysis of Spring Geometry

**Figure 1:**
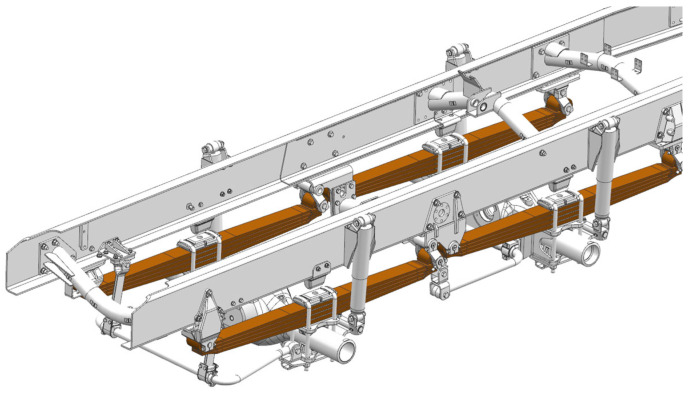
View of the geometry of the front part of the frame with the attached suspension.

In four-axle vehicles, the front wheelbase is determined by the length of the spring and the distribution of the frame of the components necessary for the proper operation of the car. This all affects the nature of the car’s operation. From the point of view of the fatigue life of the spring as well as its performance during driving, it is good if the length of the spring is as long as possible, even up to 2000 mm. However, the aforementioned limitations shorten it considerably. In the analyzed vehicle, the front wheelbase was 1700 mm, and the length of the springs was only 1600 mm. Figure 1 shows a view of the front suspension geometry of a four-axle car with semi-elliptical springs.

**Figure 2:**
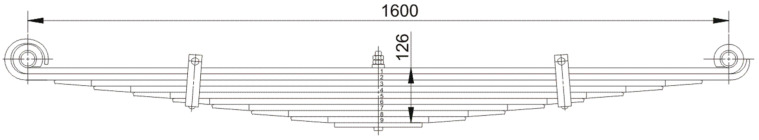
Dimensions of the tested spring.

**Figure 3:**
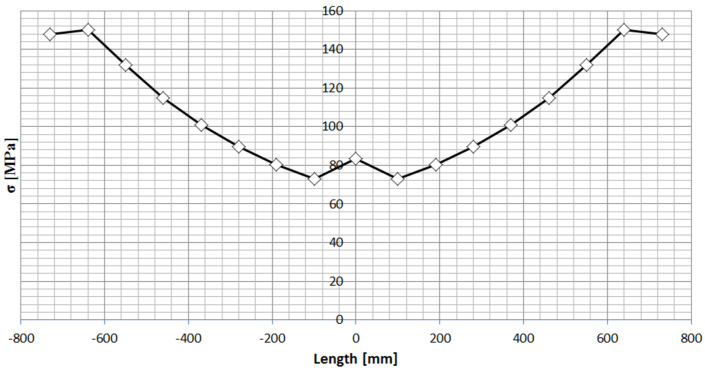
Stress distribution along the length of the spring at maximum spring deflection.

The springs used in the vehicle, as mentioned earlier, are semi-elliptical springs consisting of nine leaves of fixed width (90 mm), but of variable length. Variable leaf length is a characteristic of all semi-elliptical springs. The length of the spring, defined as the distance between the front and rear eyes when the spring is straightened, is 1600 mm, while the thickness of each leaf is 14 mm (Figure 2). A characteristic feature of the semi-elliptical spring used is that it has a large bending index (in the middle part Wx = 238.14 cm^3^), so the theoretical stress distribution due to maximum deflection is relatively small (Figure 3).

**Figure 4:**
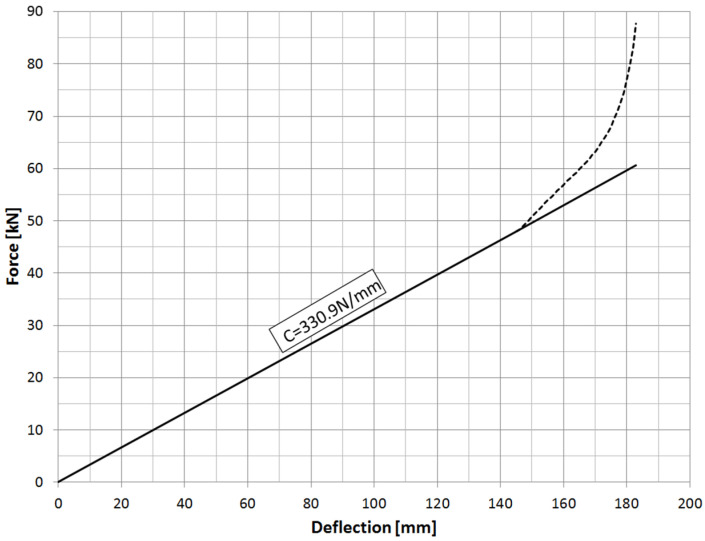
Stiffness of the spring.

The amount of deflection of the spring is determined by its stiffness, which in the case of semi-elliptical springs is linear over the entire operating range (Figure 4). As demonstrated in [14], this stiffness increases by approximately 20% when the leaf spring is clamped to the drive axle housing using U-bolts. In this case, it will be approximately 400 N/mm. The maximum deflection due to the suspension geometry is 180 mm. To ensure the safe operation of the spring in cars where suspension on springs is used, rubber buffers are installed on the frame stringer or in the center section of the spring [15], whose task is to reduce the maximum deflection of the spring, and to transfer the energy of deflection of the spring to the structural components of the frame.

**Figure 5:**
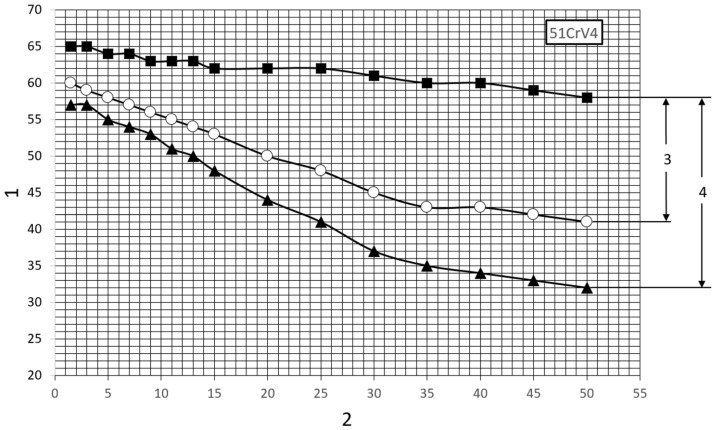
The hardenability band of 51CrV4 steel: 1—HRC hardness, 2—distance from hardened face of test specimen in mm, 3—HH grade (narrowed band), 4—H grade [8a].

In order to ensure that the springs are sufficiently resilient over the entire working range, they are made of spring steel, which has good strength properties and the ability to undergo heat treatment, as well as high fatigue strength. The leaves of the analyzed spring were made of 51CrV4 chrome vanadium steel with the elemental content shown in Table 1. Heat treatment of this steel includes hardening and high tempering. The recommended parameters for this treatment and the mechanical properties after tempering are summarized in Table 2 and Table 3. This steel is used for heavily loaded elastic components. It is characterized by a low tendency to decarburize during heat treatment procedures and high hardenability, so it can be used for parts with large cross-sections. The hardenability band of the analyzed steel is illustrated in Figure 5. The maximum dimension of the flat bar of the analyzed steel, at which there will be 80% martensite in the core after quenching in oil, is 39 mm [16]. In order to provide individual leaves with the right structure, each is rolled at the production stage from a flat bar of greater thickness, thus ensuring that the structure is properly and evenly crushed along the entire length of the flat bar. In the case under review, flat bars of type B section according to DIN 4620 [17] were used, and their initial thickness was 22 mm.

**Figure 6:**
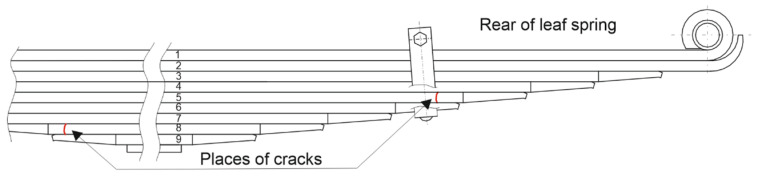
Locations of cracks in leaf springs.

**Figure 7:**
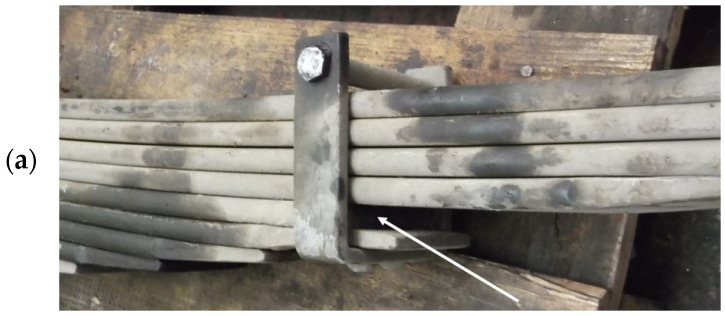

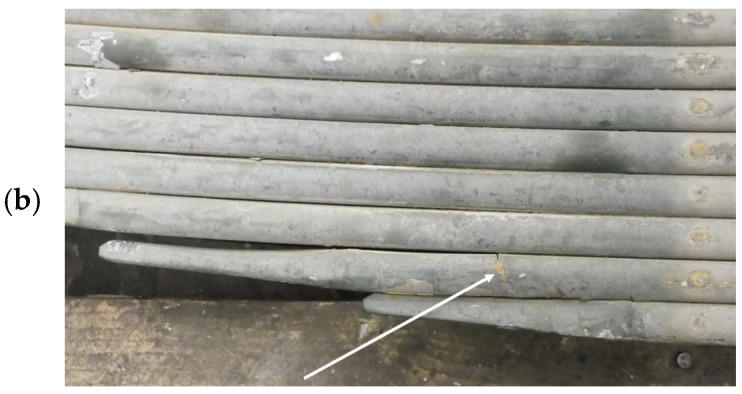
Location of spring breakage (**a**) leaf #5—swing side—rear (**b**) leaf #8—fixed side—front.

The analyzed spring was subjected to operational tests under real conditions. During the tests, the vehicle was loaded to its maximum unladen weight (8 tons/axle) and traveled over roadless and bumpy terrain, with average speeds reaching 25–30 km/h. As a result of the operation, the No. 5 and No. 8 leaves were damaged. Figure 6 shows where the damage occurred, while Figure 7 shows the broken leaves.

The cracking of both leaves eliminated the possibility of further operation of the vehicle, so it was important to determine the reasons for the excessively low service life of the spring, which amounted to about 1500 km of continuous off-road driving.

### 2.2. Metallographic Studies

**Figure 8:**
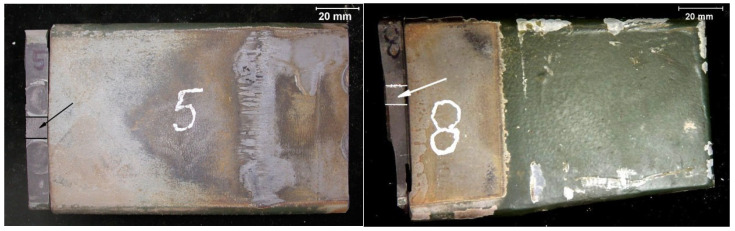
Specimen collection point.

In order to find an answer regarding the causes of the fracture of the two leaves of the semi-elliptical spring, a series of experimental tests was performed on material taken from the damaged leaves No. 5 and 8. The first stage of the study was metallographic analysis to check the quality of the heat treatment performed. The correctness of the execution of the thermal process is incredibly important in components such as leaf springs, as they are most vulnerable to damage during operation. Manufacturers of such components are required, through relevant standards, to provide the structure of the material with the required hardness, grain size, decarburization, and other characteristics that increase its fatigue life. For each of the damaged leaves, one sample slice was taken from the breakage site. The location from which the samples were taken is shown in Figure 8.

The following experimental techniques were used in the investigations:

- stereoscopic microscope: SMT-800
- scanning electron microscope: JEOL JSM 6610A equipped with an EX-230 probe
- light microscope: OLYMPUS GX51.

**Figure 9:**
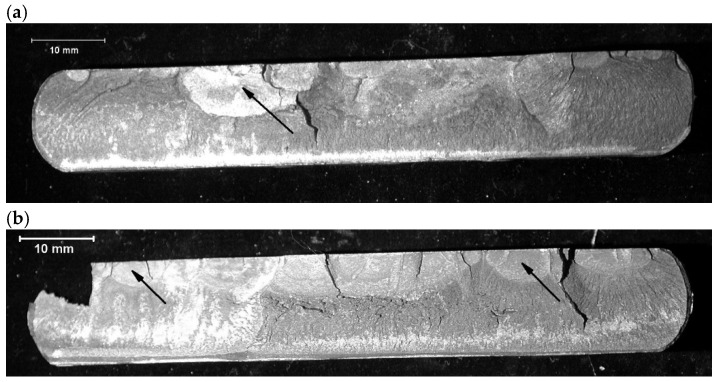
Instantaneous brittle fracture—marked fatigue fracture (**a**) leaf #5 (**b**) leaf #8.

**Figure 10:**
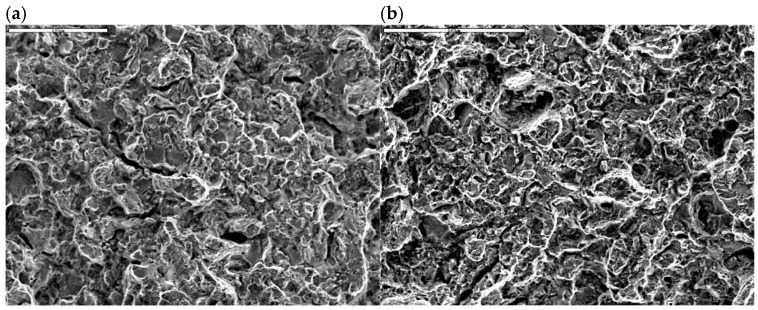
Instantaneous, brittle fracture with numerous microcracks. A magnified section of the area shown in (**a**) leaf #5, (**b**) leaf #8. Scanning Microscopy.

Fractographic studies of the fracture of the spring leaves were carried out with the unaided eye and with an SMT-800 stereo microscope, and using a JEOL JSM 6610A scanning electron microscope with an EX-230 probe, along with image acquisition. The fracture of leaf No. 5 shows a typical fatigue macrorelief (Figure 9a). The crack was initiated by operational fatigue on the tensile side of the leaf and propagated over a relatively small fatigue zone, followed by a final overload region. Numerous secondary microcracks are visible along the boundaries of structural elements in the fatigue region (enlarged area in Figure 10a). Locally, fatigue striations, characteristic of cyclic loading, can be distinguished on the fracture surface, which confirms the fatigue mechanism of crack propagation. The relatively small size of the fatigue zone indicates high nominal stresses. The calculated safety factor, determined from the ratio of the area of the entire fracture to the area of the final overload region, is 1.13. The literature states that the safety coefficients used in vehicle suspensions should be in the range of 1.3 to 1.7, indicating that the coefficient used is too small [13].

The fracture of leaf No. 8 is similar to that of leaf No. 5. A fatigue fracture is observed on the tensile side, with a clearly visible final overload region occupying most of the cross-section (Figure 9b). As in leaf No. 5, the fatigue crack was initiated on the tensile surface and propagated into the cross-section under cyclic loading. Figure 10b shows magnified SEM images of the fatigue region of leaf No. 8 with numerous secondary microcracks and locally visible fatigue striations.

The next stage of the work was observation of the fracture using an OLYMPUS GX 51 light microscope. These observations were aimed at revealing the microstructure of the analyzed spring leaves and determining their correctness. The leaf surface layers were analyzed in detail to determine the presence of a decarburized layer. The results of the observations were compared with the recommendations set by the relevant standard, according to which:

- The spring leaves, after shaping, should be subjected to heat treatment, which consists of hardening and tempering;
- The microstructure of spring leaves should be formed by highly tempered martensite (fine or medium needle-like), without traces of superheating, with no visible grain boundaries at ×500 magnification;
- Leaf decarburization should not exceed 0.3 mm from the surface [17].

**Figure 11:**
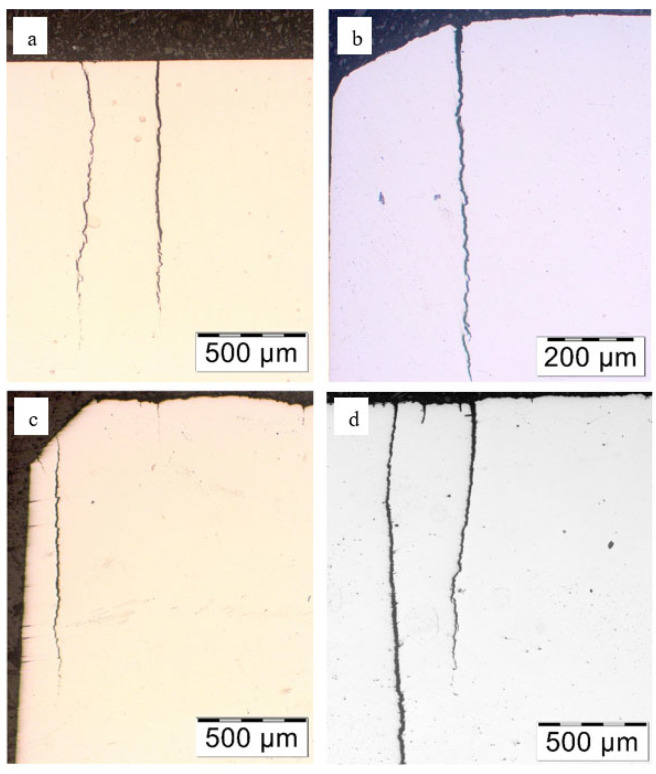
Cracks on the stretched side of the leaves: (**a**) leaf #5, magnification ×50; (**b**) leaf #5, magnification ×100; (**c**) leaf #8, magnification ×50; (**d**) leaf #8, magnification ×50; unetched state.

First, observations were made in the unetched state. Numerous cracks were observed on the tensile side of the leaves (top) (Figure 11). The depth of the largest ones was about 1 mm in leaf No. 5, while in leaf No. 8 it was several mm. No cracks were registered on the compression side of leaf #5, while single cracks occurred on the compression side of leaf #8, but their depth did not exceed 20 µm.

**Figure 12:**
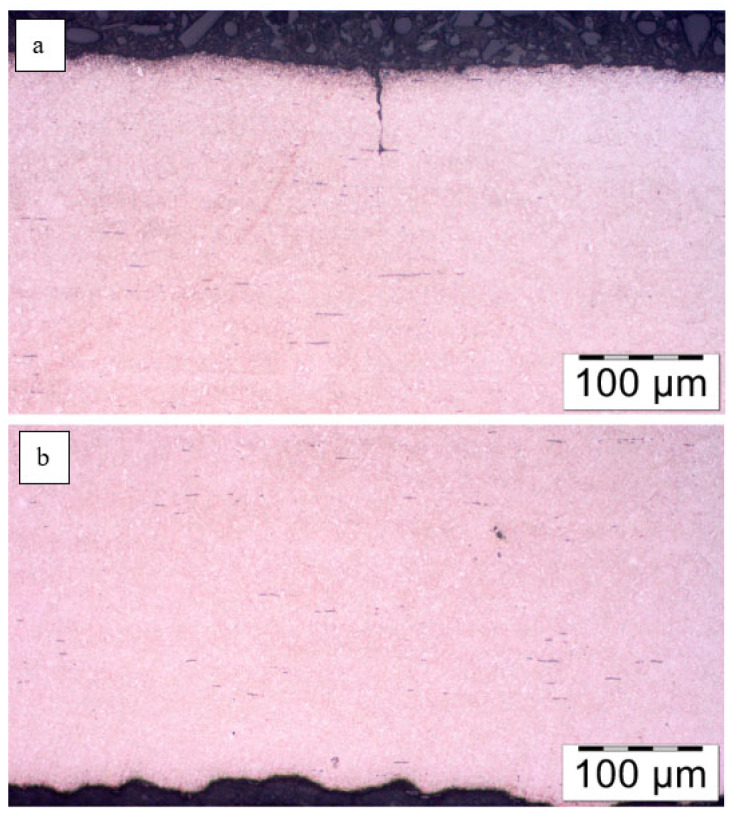
Microstructure of the surface layer of leaf #5: (**a**) tensile side, magnification ×200, (**b**) compression side, magnification ×200; nital etching.

This was followed by images of microstructures in the etched state. No changes in microstructure were observed on the surface layer of the No. 5 leaf, both on the tensile and compression sides, with respect to the core (Figure 12).

**Figure 13:**
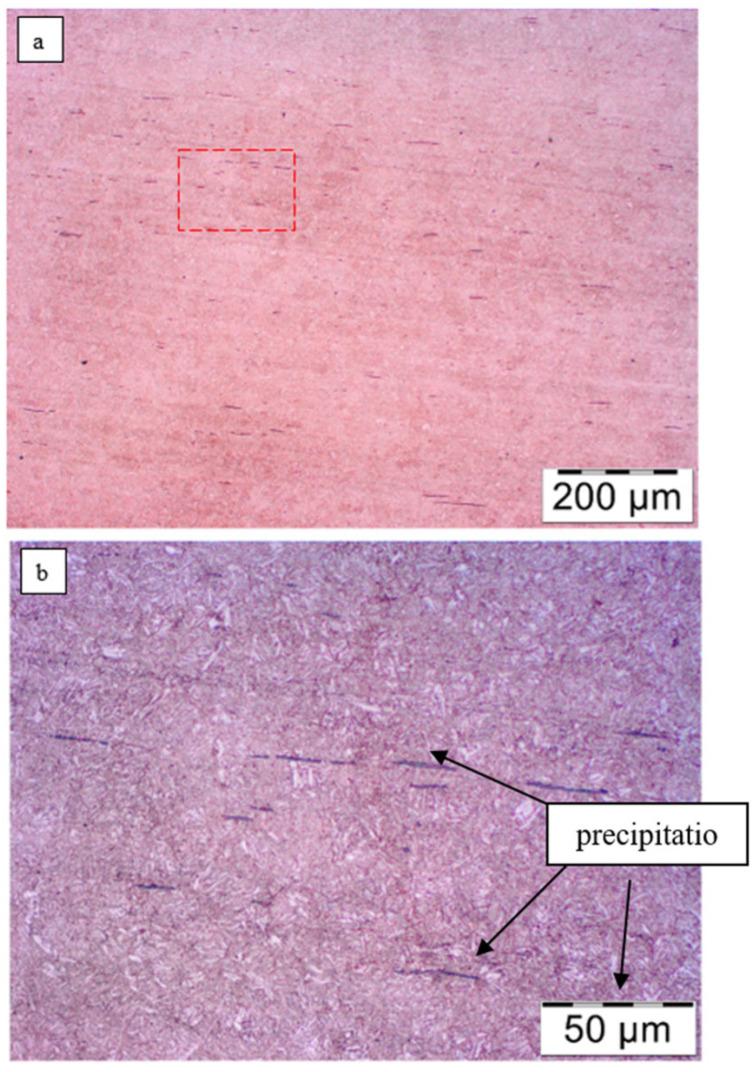
Microstructure of the core of leaf No. 5: (**a**) magnification ×100; (**b**) enlarged area marked in subsection a, area ×500; nital etching.

Numerous longitudinally shaped precipitates formed in the process of rolling the steel were observed throughout the samples. It is likely that these are non-metallic inclusions of sulfur compounds, as they tend to be most abundant in steels (the most common precipitate is MnS—manganese sulfide). Their abundance indicates the high degree of contamination of the steel tested. Figure 13 shows the microstructure of the sample core with visible longitudinal precipitates. The longest precipitates are about 100 µm.

The microstructure of the steel is formed by the tempering sorbite (highly tempered martensite), so it is a postmartensitic structure. Light aciform precipitates of supersaturated ferrite can be seen. This suggests that heat treatment procedures were carried out on the analyzed steel after the leaf was shaped (quenching + high tempering). The structure of the tempering sorbite is favorable for spring steel, as it provides it with adequate elasticity and lack of susceptibility to plastic deformation while maintaining adequate impact strength.

**Figure 14:**
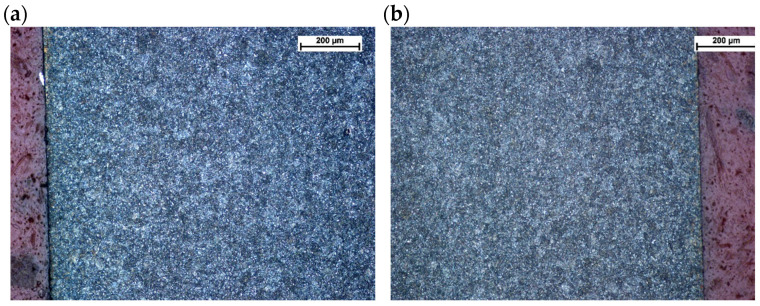
Leaf surfaces without signs of decarburization (**a**) tensile side, (**b**) compression side. Light microscopy. Mi1Fe etching.

The surface of the sample was also checked for signs of decarburization. Figure 14 shows a view of the surface of leaf No. 5, which showed no signs of decarburization of the surface layer.

**Figure 15:**
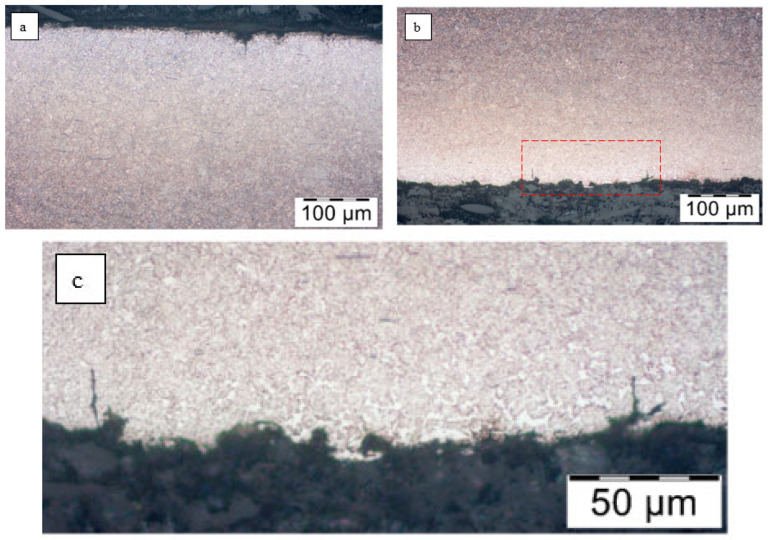
Microstructure of the surface layer of leaf No. 8: (**a**) tensile side, magnification ×200; (**b**) compression side, magnification ×200; (**c**) enlarged area marked in subsection b, magnification ×500, nital etching.

**Figure 16:**
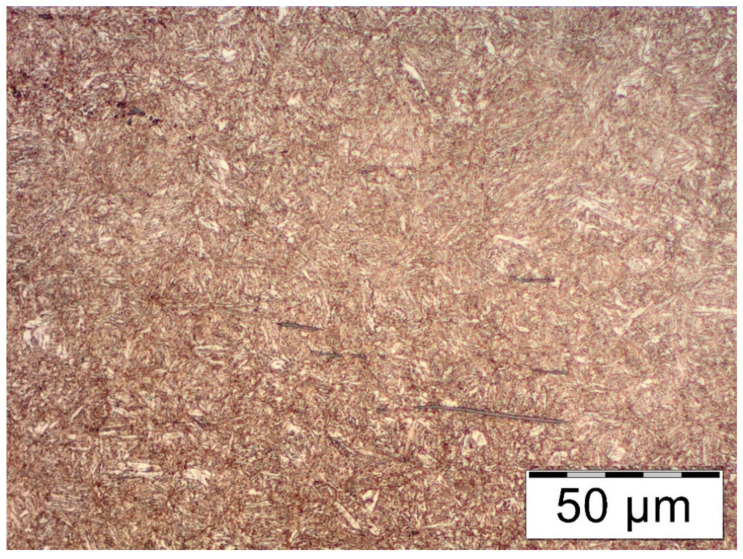
Microstructure of the core of leaf #8, magnification ×500, nital etching.

A similar analysis was performed for the second leaf. Figure 15 shows the microstructure of the top layer of leaf #8 at the fracture site. No changes in microstructure were observed on the tensile side (Figure 15a), while single small precipitates of light phases were noted on the compression side (Figure 15b). The maximum depth of their occurrence does not exceed 50 µm. At ×500 magnification, martensite platelets are clearly visible (Figure 15c and Figure 16).

The core microstructure of the No. 8 leaf is formed by a tempering sorbite, similar to that of the No. 5 leaf. Characteristic packets of martensite platelets can be seen. Numerous elongated non-metallic inclusions were observed on the background of the sorbite (Figure 16).

The microstructure of the analyzed leaves meets the assumptions of the standard [6] regarding morphology (highly tempered martensite), but indicates a slight overheating of the steel during austenitization. For low-alloy steel enriched with vanadium addition, it is possible to obtain a finer structure after thermal operations.

**Figure 17:**
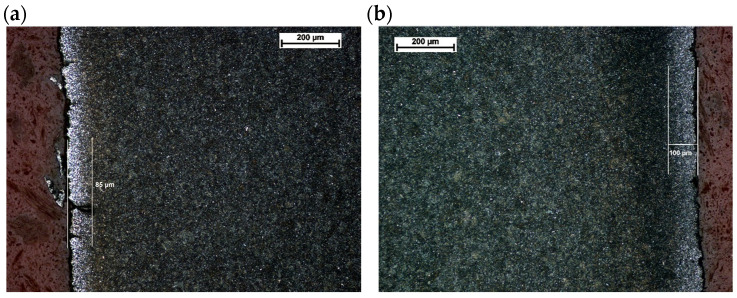
Decarburized layer with microcracks (**a**) tensile side, (**b**) compression side.

In leaf No. 8, a small decarburized layer about 0.1 mm deep was noted on both surfaces (compression and tension) (Figure 17).

### 2.3. Hardness Measurements

Microhardness measurements to determine the presence of a decarburized layer near the surface were made at 10 points from the surface deep into the material. The first measurement point was placed at a depth of 50 µm, while the last was placed at 550 µm. Two series of measurements were made on the tensile and compression sides for each specimen. In addition, 8 leaf core microhardness measurements were taken in each sample. The distribution of microhardness is shown in the charts with horizontal lines marking the range of hardness values recommended by the standard for leaf springs: the hardness of leaf springs should be in the range of 363 ÷ 460 HB, i.e., 382 ÷ 490 HV [18].

Unfortunately, current standards do not specify the expected hardness of spring steels after heat treatment procedures [9], but only process parameters and other mechanical properties. Only the standard for springs mentions the hardness of the leaf after heat treatment. In order to compare the obtained hardnesses of the core of the studied steel grade, we used the results of the authors who analyzed the properties of 51CrV4 steel after heat treatment [16,17]. The results of their hardness measurements with the parameters of the processes used are presented in Table 4.

Table 5 shows the results of leaf core microhardness measurements. The obtained values are within the limits of the values recommended by the standard for spring leaf requirements: 363 ÷ 460 HB (382 ÷ 490 HV) [19]. Comparing the results of the measurements with the hardnesses of this steel grade after heat treatment (Table 4), it can be seen that the values obtained are higher, indicating the use of lower tempering temperatures than in the heat treatment carried out by the cited authors.

**Figure 18:**
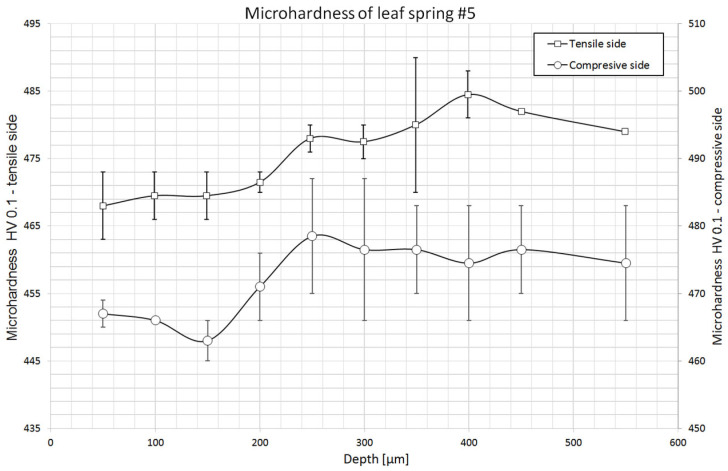
HV 0.1 microhardness distribution of leaf #5.

No decrease in microhardness was recorded on either the tension or compression side of leaf No. 5, indicating the presence of a decarburized layer (Figure 18). In both cases, the microhardness increases in the direction of the core, but its differences throughout the measurement section are small, on the order of 15 HV.

**Figure 19:**
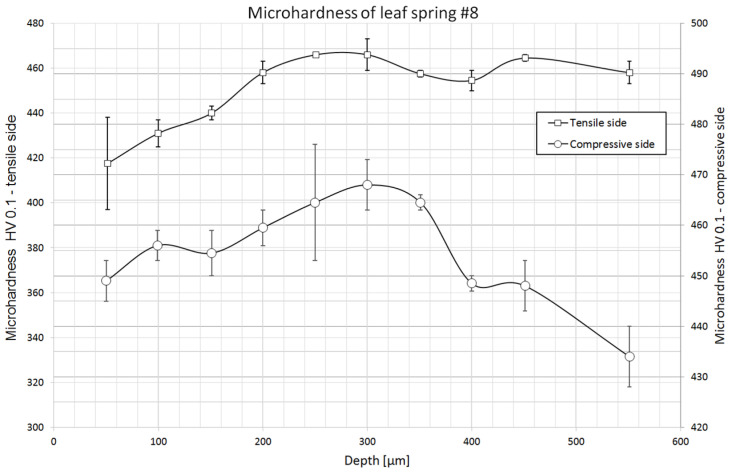
HV 0.1 microhardness distribution of leaf #8.

On the tensile side of the No. 8 leaf, one can see a clear increase in hardness with increasing depth. The difference in hardness between the surface and the core is about 50 HV (Figure 19), which would indicate a slight decarburization of the surface. The smallest recorded hardness value at the tensile surface meets the minimum requirements of normative recommendations [19]. On the compressed side of the leaf, the distribution of hardness indicates the absence of decarburization, as the differences in hardness values are small, plus there is no clear upward trend toward the core. Light phases were observed during microscopic observations, but their depth and frequency were small enough that their presence is not apparent on the microhardness distribution.

*Summary of the Research Part*

Observations with a light microscope revealed a postmartensitic structure—most likely, the steel had been heat-treated by quenching and tempering. Such a structure was observed throughout the element’s cross-section. Its morphology is normal, but at ×500 magnification, martensite platelets can be clearly distinguished, indicating overheating of the steel during austenitizing. Core hardness is relatively high compared to data from literature sources. Its value and the shape of the martensite platelets suggest that the tempering temperature was lower than that recommended by the standard for heat treatment of spring steels [20], but it meets the requirements of the range of hardness values of steels for leaf springs. Tests revealed no significant structural defects near the surface of the components. The only anomaly is the presence of light phases near the compressed surface of the No. 8 leaf, but their amount is insignificant. The main inconsistency noted was the large amount of longitudinal non-metallic precipitates on the matrix of tempered martensite.

Based on the microhardness distributions deep into the material, no decarburized layer was found. Only on the tensile side of the No. 8 leaf was there a decrease in hardness near the surface, but it is relatively small. The results of these measurements suggest that appropriate protective atmospheres were used during heat treatment to prevent surface decarburization. No differences in the microhardness distributions near the surface of the leaves were noted to clearly suggest the use of peening on the tensile side of the leaves, but the difference in the regularity of the surface indicates that it probably took place. On the tensile side of the leaves, the surface appears to be more uniform and less rough, which may be a direct result of the finishing surface treatment, but no cold hardening of the material was observed in the hardness values.

### 2.4. Numerical Analysis of Spring Geometry

**Figure 20:**
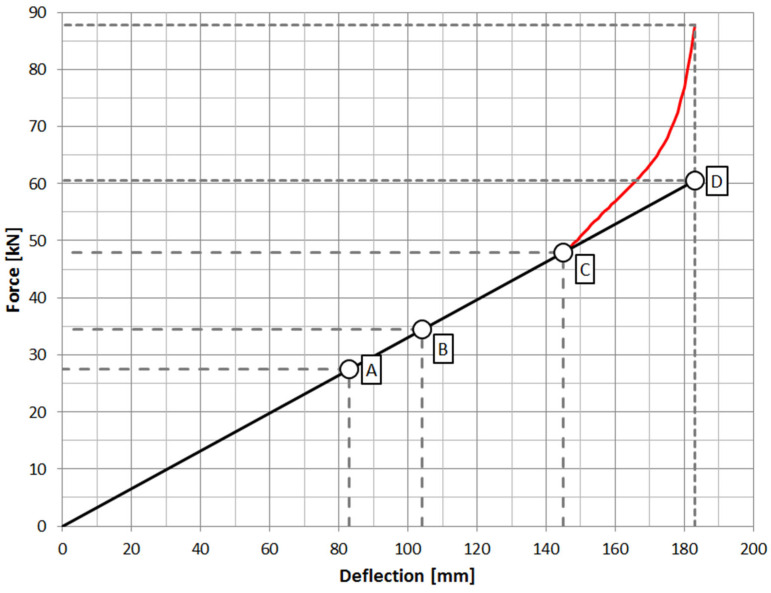
Characteristic points for the work of the spring: A—straightened spring, B—maximum load on the axle of the vehicle, C—beginning of the work of the buffer with the spring, D—maximum deflection of the spring.

The next stage of the work carried out was the determination of leaf stress levels, particularly in the zone of damage that had occurred. A numerical model of the spring was developed using the finite element method, which was then subjected to loads resulting from the performance characteristics of the spring at its boundary points marked in Figure 20.

**Figure 21:**
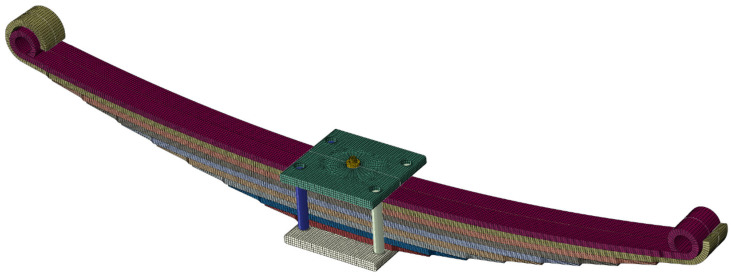
Numerical model of the leaf spring.

A numerical model of the leaf spring was made using HEXA 8, HEXA 20, and TETRA 10 volume elements [21,22]. The leaf springs were made from HEXA 20-type elements. An analysis of the convergence of the results was performed on the basis of which the number of elements over the leaf’s thickness was selected. The length of the finite element was no more than 5 mm, which is in accordance with the recommendations given by Savaidis [23]. The model considers how the spring is connected to the truck’s drive bridge. Friction occurring between contacting elements was taken into account. Also taken into account are the forces resulting from the tension of the stirrups and the central bolt that fastens the individual leaves together [24]. Figure 21 shows a view of the numerical model. The calculations were performed in ABAQUS.

**Figure 22:**
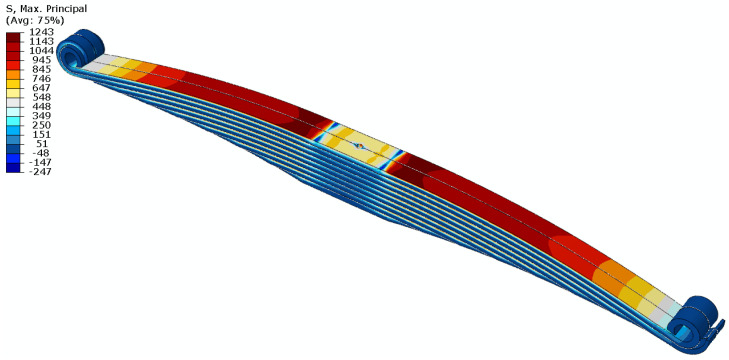
Principal stress contours s1 in MPa in the spring.

The distributions of principal stresses at the deflection of the spring at its characteristic points are shown in Figure 22.

**Figure 23:**
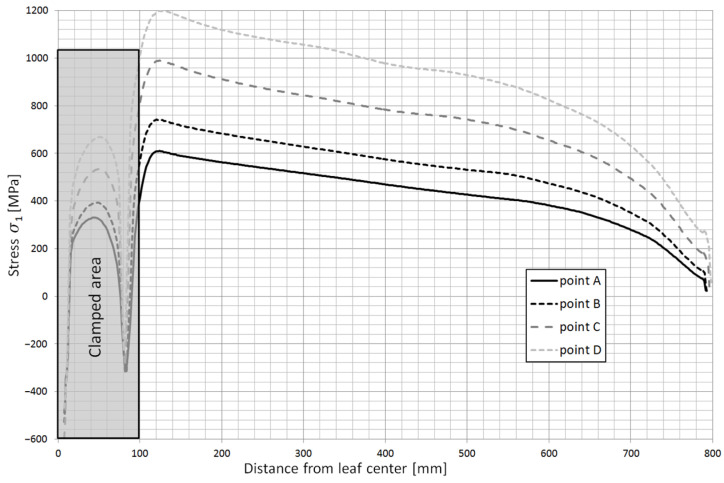
Stress contours along the main leaf (No. 1) at characteristic points of spring operation.

**Figure 24:**
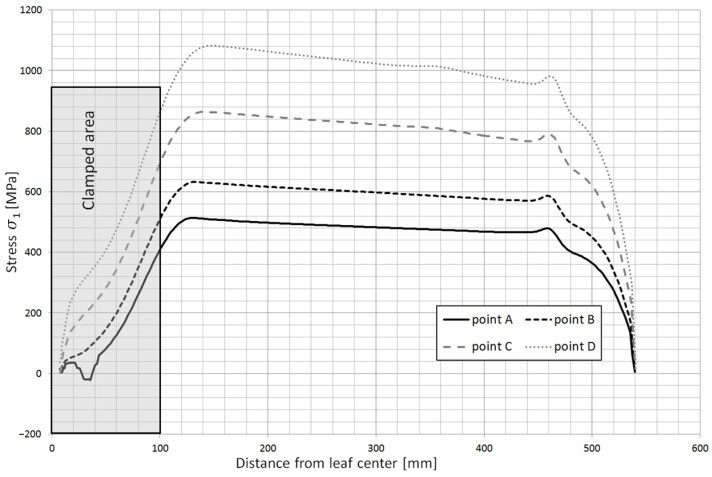
Stress contours along leaf no. 5 at characteristic points of spring operation.

**Figure 25:**
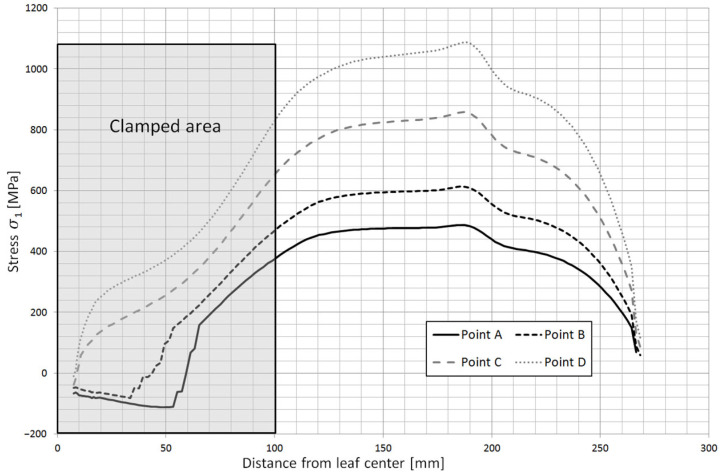
Stress contours along leaf no. 8 at characteristic points of spring operation.

Based on the obtained calculation results, the stress contours along the individual leaves were obtained. Figure 23 shows the stress contours in the main leaf for each characteristic point of the spring, while Figure 24 and Figure 25 show stress plots along the length for the same points but for leaf 5 and 8.

**Figure 26:**
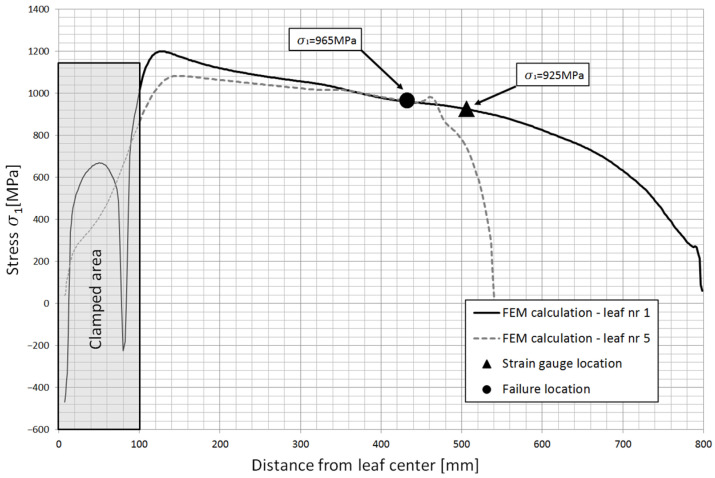
Comparison of principal stress contours in leaf #5 obtained numerically.

**Figure 27:**
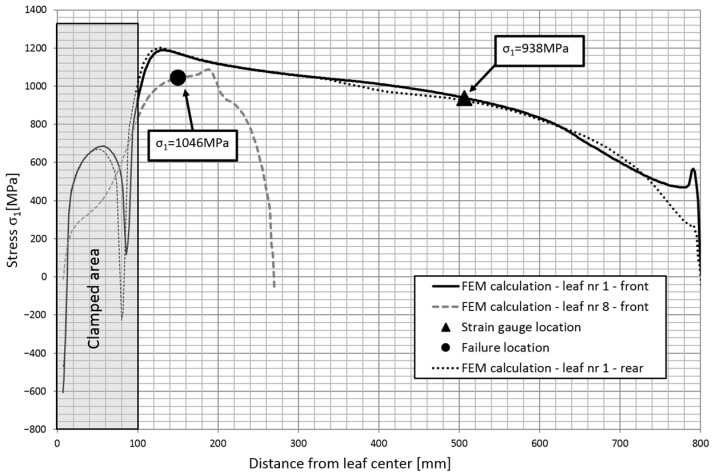
Comparison of principal stress contours in leaf #8 obtained numerically.

Based on the numerical analysis, the magnitude of the strain in the zones where the leaves underwent fatigue fracture was identified. Figure 26 and Figure 27 show the principal stresses σ1 in the leaves at the maximum deflection arrow of the spring. The contours are shown for the leaves that fractured, and then compared to the stress contours of the main leaf on which the strain gauges were attached. The location of the cracks and the location of the measurement points are also marked. The stress difference between the T4 point and the fracture zone of the No. 5 leaf is about 5% over the entire working range. In contrast, the stress difference between the T11 point and the fracture zone of the No. 8 leaf is about 10%.

### 2.5. Experimental Tests

Determining the cause of spring failure based on metallographic examination alone is complicated when no clear answer can be found. Therefore, in addition to the strength analysis of the spring using the finite element method, additional tests were performed on an identical spring installed on a truck that was traveling at similar speeds under the same road conditions.

**Figure 28:**
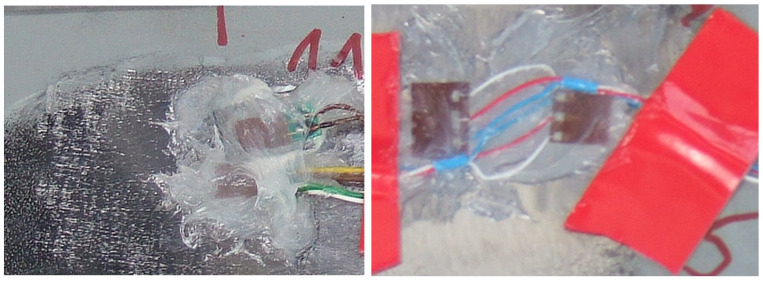
Installed measuring points.

**Figure 29:**
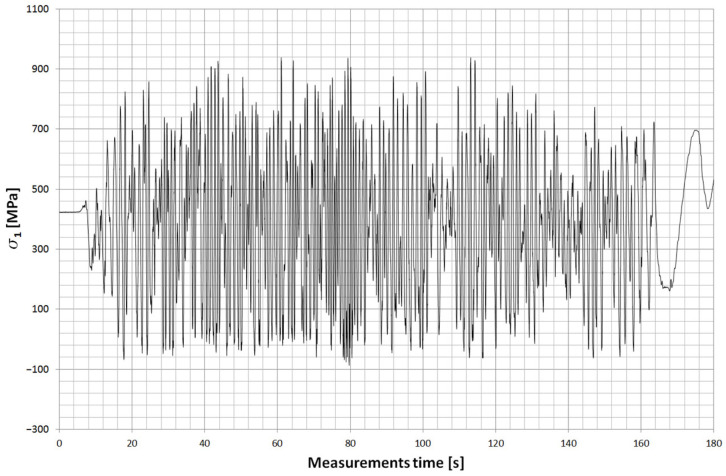
Example of the stress contours in the spring at T11 (in front of the axle).

**Figure 30:**
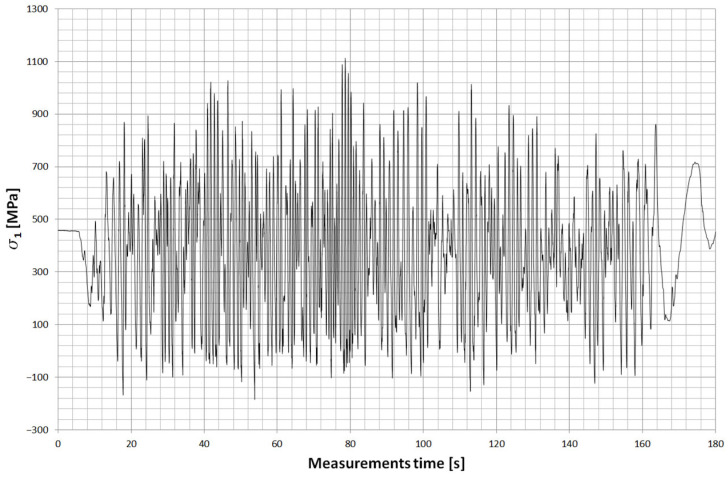
Example of the stress contours in the spring at T4 (behind the axle).

Two strain gauge points were placed on the main leaf of the spring. The first was located about 505 mm (Figure 28) from the axis of the drive bridge and was installed behind the axle in the rear direction. The other, on the other hand, was at a distance of about 150 mm and was installed in front of the axle in the forward direction of the car. Both strain gauges were installed on the upper surface of the leaf, which is mainly stretched during the life of the spring. Figure 29 and Figure 30 show examples of stress contours in the main plume recorded during a drive along one of the survey sections of about 1.2 km [25,26,27]. As can be seen from the obtained contours, the maximum stresses reach values for the T4 point of about 1000 MPa, while for the T11 point (the point in front of the axis), in some cases, exceed 900 MPa. At both measurement points, the minimum stresses reach a negative value, indicating that the wheel axle is being relieved during operation, or large vertical displacements of the frame are occurring, causing the spring to be relieved.

For both measurements, the average value of stresses is, respectively:

s_T4_ = 375 MPa, s_T11_ = 378 MPa.

Stress range:

Ds_T4_ = 960 MPa; Ds_T11_ = 1183 MPa.

The obtained measurement results were compared with those obtained by the finite element method using a scaling factor for each measurement point to be able to obtain stress values in the leaf fracture zone.

**Figure 31:**
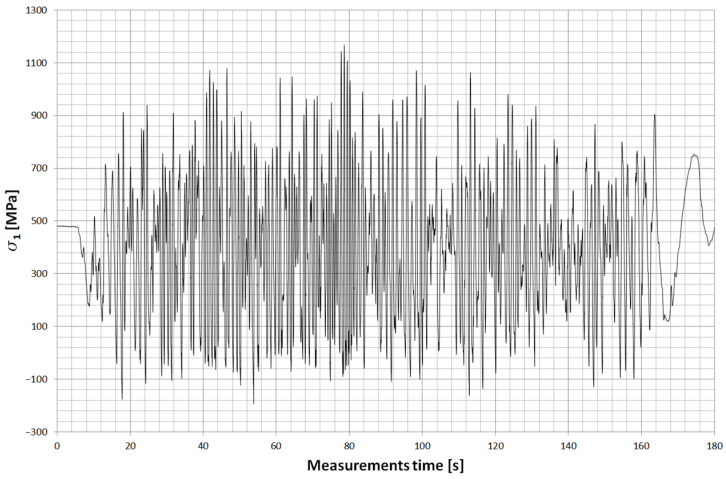
Stress contour in the fracture zone of leaf #5.

**Figure 32:**
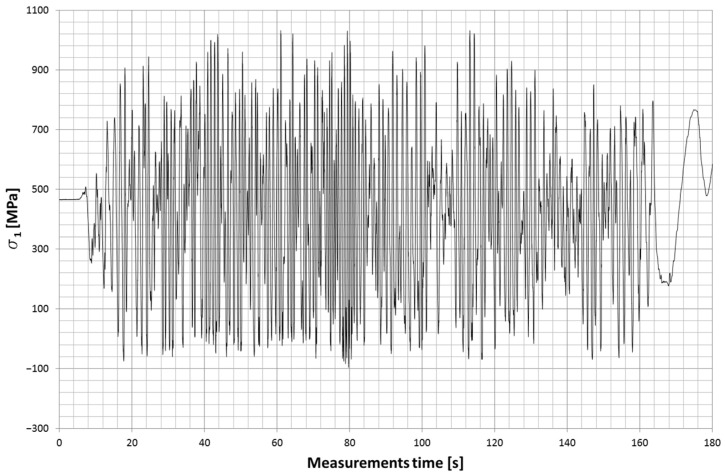
Stress contour in the fracture zone of leaf #8.

The stress contour for the fracture zone of leaf #5 is shown in Figure 31, while the stress contour for the fracture zone of leaf #8 is shown in Figure 32.

**Figure 33:**
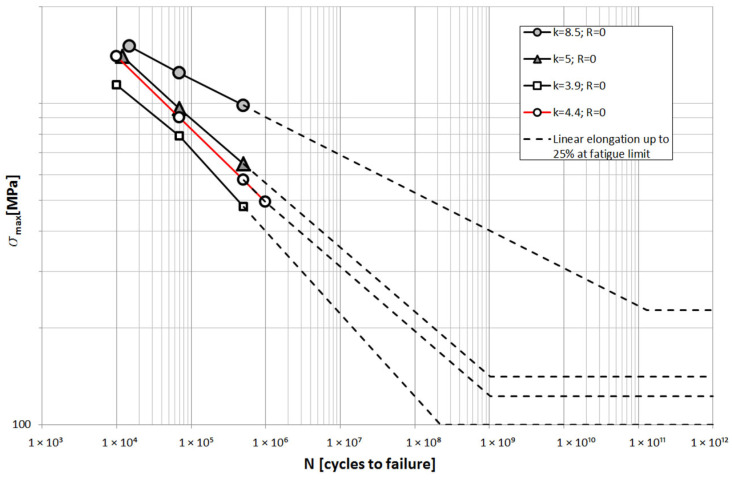
Assumed fatigue characteristics of the spring material [21].

The obtained stress variation contours were used for further analysis in an attempt to answer the question of what the fatigue life of such a spring would be at such high levels of strain. Due to the lack of accurate material tests of the steel from which the spring was made, data for fatigue calculations were taken from the literature [23,28,29]. Fatigue analysis was performed for the 51CrV4 steel mentioned earlier. A total of four fatigue characteristics of the same grade of steel were adopted, for which different parameters of the spring manufacturing technology were used. They differ in heat treatment parameters, finishing technology, etc. More on this subject is explained in [30,31]. The heat treatment and finishing of the tested spring is similar to the material, whose fatigue curve slope factor k is k = 4.4. It was also assumed that the fatigue life of steel decreases to a value of 25% of the fatigue limit at low-amplitude cycles for which the stress range is below the fatigue limit [32,33]. Figure 33 shows the adopted fatigue characteristics of the leaf.

The number of cycles was estimated for the measured measuring section using the rain-flow method. These values were then multiplied by a distance of 1000 km, and the fatigue life was determined. Table 6 shows the obtained values of the number of cycles, fatigue life for each fatigue curve.

## 3. Summary and Conclusions

A full spectrum of experimental and numerical studies of the components of a semi-elliptical spring composed of nine leaves of variable length was performed. The purpose of the research conducted was to find the reasons for the excessively rapid degradation of two leaves. The spring was operated during continuous off-road driving, the surface of which was heterogeneous, having many obstacles both longitudinally and laterally, with low ground stability. During the tests, the vehicle was loaded to a total empty weight of 32,000 kg.

Visual tests of the fracture of the two broken leaves were conducted. Metallographic tests were then performed to check the microstructure of the material for the correctness of the heat treatment technology. The data obtained were compared with those presented in the relevant standards. The microhardness of the specimen’s fracture was then measured to estimate whether a finishing process had been applied.

The next stage of the work performed was to conduct experimental tests on the target car, loaded with the same load and equipped with the same type of springs. Since the location of the cracks in the leaf springs makes it impossible to install strain gauges at this point, they were installed on the main leaf, and then the difference between the stresses occurring in the leaf fracture zone and the measuring point was determined using the finite element method. Based on this, the load values of both leaves were obtained. Then, based on the results obtained using the rain flow method, the number of cycles that occurred during the passage through the survey section was estimated. These values were compared to a 1000 km section, and then the fatigue life of the spring was estimated for 51CrV4 steel made using different heat and finishing technologies and parameters.

From the experimental study, it was found that the fatigue life of the spring was too short:

- Improperly matched spring to the operating load conditions of the car;
- Excessive strain on the spring during static loading—spring inflection;
- Inadequacy of heat treatment parameters to meet the temperature requirements for hardening and tempering of spring steels;
- Lack of properly executed finishing technology;

It was estimated that the fatigue life of the cracked spring for the measured loads and selected material parameters would be no more than 1400 km. This result is comparable to the actual number of kilometers the vehicle traveled before the spring leaves broke.

The presented fatigue analysis for a spring made of the same material but for different heat treatment and finishing conditions showed that it is possible to significantly increase the fatigue life of the spring. The condition is the use of appropriate technology. The requirement is to use an appropriate technology, which is described in more detail in articles [30,31].

## Figures and Tables

**Table 1:** Approximate chemical composition of 51CrV4 steel according to the European standard PN-EN 10089: 2005 [16].

Element Content [%]						
C	Si	Mn	Cr	V	Smax.	Pmax.
0.47 ÷ 0.55	≤0.40	0.70 ÷ 1.10	0.90 ÷ 1.20	0.10 ÷ 0.25	0.025	0.025

**Table 2:** Approximate mechanical properties of tempered test specimens taken from 51CrV4 steel [16].

R_p0.2 min._ [MPa]	R_m_ [MPa]	A_min._ [%]	Z_min._ [%]	Impact Strength at 20 °C Ku Min. [J]
1200	1350 ÷ 1650	6	30	8

**Table 3:** Recommended heat treatment parameters for 51CrV4 steel [16].

Hardening Temperature	Hardening Medium	Tempering Temperature
850 ± 10 °C	oil	450 ± 10 °C

**Table 4:** Hardness of 51CrV4 steel after heat treatment based on literature [19,20].

No.	Hardening	Tempering	HV Hardness
1 [19]	870 °C/10’	475 °C/1 h	446
2 [20]	860 °C/30’	450 °C/2 h	434
Sample no. 5	-	-	475 ± 11
Sample no. 8	-	-	467 ± 9
Guidelines of the standard	-	-	382 ÷ 490

**Table 5:** Results of HV 0.1 leaf core microhardness measurements.

Core Microhardness HV 0.1	
leaf #5	leaf #8
475 ± 11	467 ± 9

**Table 6:** Data obtained after fatigue analysis.

	m = 3.9		m = 4.4		m = 5		m = 8.5	
	P5 Leaf	P8 Leaf	P5 Leaf	P8 Leaf	P5 Leaf	P8 Leaf	P5 Leaf	P8 Leaf
Total number of cycles N [-]	326,400	441,600	326,400	441,600	326,400	441,600	326,400	441,600
Number of cycles considered for fatigue life estimation [-]	165,600	172,800	153,600	158,400	144,000	154,400	114,400	124,000
Degree of fatigue life depletion D [%]	**119**	**137**	70	81	50	58	5	6
Predicted number of kilometers until failure [km]	841	728	1429	1235	1981	1713	18,523	16,498

## Data Availability

The original contributions presented in this study are included in the article. Further inquiries can be directed to the corresponding author.
